# Dentin bond strength of bioactive cement in comparison to conventional resin cement when photosensitized with Er,Cr:YSGG Laser

**DOI:** 10.12669/pjms.36.2.1284

**Published:** 2020

**Authors:** Fahad Alkhudhairy, Sulieman S. Al-Johany, Mustafa Naseem, Mohammed Bin-Shuwaish, Fahim Vohra

**Affiliations:** 1 Fahad Alkhudhairy, Department of Restorative Dental Sciences, College of Dentistry, King Saud University, Riyadh, Saudi Arabia; 2 Sulieman S. Al-Johany, Department of Prosthetic Dental Sciences, College of Dentistry, King Saud University, Riyadh, Saudi Arabia; 3 Mustafa Naseem, Department of Community Dentistry, Dow International Dental College. Karachi Pakistan; 4 Mohammed Bin-Shuwaish, Department of Restorative Dental Sciences, College of Dentistry, King Saud University, Riyadh, Saudi Arabia; 5 Fahim Vohra, Department of Prosthetic Dental Sciences, College of Dentistry, King Saud University, Riyadh, Saudi Arabia

**Keywords:** Bioactive cement, Conventional resin cement, Dental aesthetics, Er,Cr:YSGG, Shear bond strength

## Abstract

**Objective:**

The purpose of the present study was to assess dentin shear bond strength (SBS) and mode of bond failure of bioactive cement (BA) in comparison to conventional resin cement when photosensitized by Er,Cr: YSGG Laser (ECL).

**Methods:**

The present in-vitro study was carried out from March 2019 to May 2019. Sixty permanent non-carious, intact, non-fractured molars were isolated and mounted vertically in acrylic resin. Buccal surface of each molar tooth was ground, polished and surface treated with ECL. Ketac conditioner was applied on the surface washed and air dried surface. Tetric -N-Bond adhesive was applied on forty-five samples and light cured. The specimens were allocated into four groups (n=15) according to the type of cement used i.e., Calibra (C), BA, Variolink II (V) and Maxcem-Elite (ME). For SBS testing was performed using the universal testing machine. Eight samples from each group were assessed for modes of failure. Means and standard deviations were compared using analysis of variance (ANOVA) and Tukey’s post hoc test at a significance level of p < 0.05.

**Results:**

The highest mean SBS was observed in group ECL-C (21.55±3.08). The lowest mean SBS was displayed in group ECL-ME (14.25±3.55). Mean SBS values for group ECL-C (21.55±3.08) and group ECL-V (20.74±4.15) were comparable (p <0.05). Similarly, SBS values of group ECL-BA (15.48±3.62) and group ECL-ME (14.25±3.55) were comparable (p <0.05).

**Conclusion:**

Dentin surface conditioned with Er,Cr: YSGG and bonded to C and V cements exhibit favourable bond strength values.

## INTRODUCTION

Clinical success of direct and indirect restorations requires an excellent adhesive bond between tooth and the restorative material. Adequate adhesion is a product of the agents used for the cementation of restorations.^1^ Ideally, an appropriate luting cement must offer good marginal seal, optimal compressive, tensile and shear bond strength (SBS), resistance to dissolution in oral cavity, wettability and aesthetics.^2^ In contemporary dentistry, dual cure luting cements are more acceptable among clinicians for indirect bonding as they exhibit low microleakage scores, solubility, available in different viscosities, improved mechanical properties, ease of handling and compatibility with different bonding agents.^3^,^4^

Continued research in the field of materials has resulted in the introduction of bioactive (BA) resin cement for clinical use. The BA cement has the capacity to remineralize tooth-restorative interface, by exchange of fluoride, calcium and phosphate ions with saliva and tooth structure. The material stimulates mineral apatite crystal formation due to the presence of bioactive glass filler and bioactive resin matrix. According to the manufacturer, BA cement displays better mechanical properties then conventional cement, better marginal seal, is antibacterial, and is much durable with improved aesthetic properties.^5^

Bonding to dentine surface remains challenging and technique sensitive.^6^ Lately, use of Erbium, chromium-doped yttrium, scandium, gallium and garnet Er,Cr:YSGG laser (ECL) for photosensitizing the dentinal and enamel surface of the tooth has shown favourable results.^7^,^8^ ECL works at wavelength of 2780nm opening the dentinal tubules and making the surface reactive for adhesion. Laser modulation on dentin surface alters the carbonate to phosphate ratios, reduces the organic and water proportion in dentin, making the dentinal surface resistant to acid attack. This can be of great importance when luting an indirect restoration.^9^,^10^

To our knowledge from indexed literature evidence is limited in relation to bond strength of bioactive cement to ECL lased dentine. Studies assessing the influence of lased ECL dentin, show controversial outcomes when bonded to different conventional cements.^11^,^12^ It is hypothesized that dentin conditioned with ECL and bonded to BA cement will exhibit better SBS results compared to conventional dual cure cement. Therefore, the aim of the present study was to assess mode of failure and SBS of BA cement in comparison to conventional cement when dentin surface is photosensitized by ECL.

## METHODS

The present in-vitro study was permitted by the ethical committee of King Saud University with ethical approval (Ref. No. 18/0680/IRB dated October 25, 2018) project no. E-18-3345. The study followed the Check List for reporting in-vitro study (CRIS) guidelines.

Sixty permanent non-carious, intact, non-fractured permanent molars were isolated and cleaned from debris and inorganic remnants with the help of periodontal curette and scaler (Superior Instruments Co, New York, USA). Before beginning the experiment, in accordance with Human Tissue Act, 2004 the teeth were stored in 10% formalin buffer solution for 48 hours and then transferred to distilled water until preparation. The present study followed checklist for reporting in vitro studies (CRIS) guidelines.

All the specimens were mounted vertically in acrylic resin (Meliodent, Kulzer, Hanau, Germany) within the segments of polyvinyl pipes (4mm radius) equal to cementoenamel junction (CEJ) revealing only the clinical crown. To homogenize dentinal depth and to expose fresh dentinal tubules the buccal surface of all molars were ground to a depth of 2mm with an area of 3mm using Isomet saw (Buehler, Illinois, USA). The surfaces were polished with a 300–500 grit silicon carbide paper (Buehler, Illinois) on a rotary polishing machine (Aropol 2V, Arotec) (250 Rpm) under water irrigation for 20 s.

Buccal dentinal surface of each specimen was conditioned by ECL (Waterlase C-100, BioLase Tech Inc., California, USA) power 4.5W and frequency 30Hz in a non-contact mode from a distance of 2mm using tip (MZ=8) for a duration of 60 Seconds. After laser procedure, the specimens were bathed in artificial saliva (NeutraSal, Orapharma, North America LLC) for 60 s. The samples were rinsed dried using compressed air removing moisture. Ketac conditioner (3M ESPE, Dental products, Seefeld-Germany) was used on the dentinal surface for 10 s and then washed with copious water for 10 s and air dried. A universal bonding agent (Tetric N-Bond Universal, Ivoclar-Vivadent) was applied and light cured (Bluephase G2, Ivoclar, Vivadent) for 10 s on 45 specimens only. Specimens were allocated into four groups (n=15) according to the type of cement (Table-I).

**Table-I:** Types of luting cement along with composition used in the study.

Product	Type of polymerization	Composition	Manufacturer
Bioactive Cement	Dual Cured	Diurethane and other methacrylates, polyacrylic acid, amorphous silica, sodium and fluoride	Pulpdent
Variolink II Aesthetic cement	Dual Cured	UDMA, TEGDMA, Self-curing initiators silicon dioxide, pigment, stabilizers, light curing initiators	Ivocalar, Vivadent
Calibra luting cement	Dual Cured	BISGMA monomer, Benzoyl peroxide Camphorquinone, coupling agent, glass filler, peroxide	Dentsply, Caulk
Maxcem Elite cement	Dual Cured	Proprietary redox initiators, photo initiators, Resin: GPDMs, DMAs Fillers: barium, fluor- aluminosilicate, and silica	Kerr, Corporation

### Group-1

BA (Activa, Pulpdent Cooperation, Watertown, Massachusetts USA) was dispensed in a polyether rubber mould which was already placed on the dentine samples using a cement plunger and was light cured (Bluephase G2, Ivoclar, Vivadent) for 20 Seconds. The moulds were removed carefully.

### Group-2

Application of Variolink II (V) (Ivocalar, Vivadent) was done by mixing both paste A (catalyst) and paste B (base), dispensed in polyether rubber mould and pre-cured for 10 Seconds. After removing excess, the cement was again cured 40 s each from two different directions.

### Group-3

Maxcem Elite (ME) (Kerr, Corporation) cement was auto mixed using a single tip and dispensed on the dentinal surface. Initially, cured for 2 s and then excess cement was removed. Then the cement was cured for 10s each from all the surfaces.

### Group-4

Calibra (C) (Dentsply, Caulk) cement was dispensed equally as base and catalyst and mixed in accordance to manufacturer instructions and applied on dentine surface within the polyether rubber mould. Initially the cement was auto-polymerized (self-cure) for three minutes and again for 40 s after removing excess.

After sample preparation, all the specimens were kept in an incubator (Memmert Universal Oven, Germany) at 37°C in a humid environment for two days. Further, the samples were thermocycled between 5°C to 60°C for 8000 cycles (Applied Biosystems, Automated Thermal Cycler (ATC), CA, USA) for 45 s each, before assessing the shear bond strength.

For SBS the specimens were tested using the universal testing machine (Instron 8500 Plus, Canton) with a cross head speed of 0.1mm/s at the dentine cement interface. The shear strength that separated the test material was calculated. Similarly, eight samples from each group were assessed for modes of failure (40X magnification) using a stereomicroscope (SZX7, Olympus, Hamburg, Germany) and classified into cohesive, adhesive and admixed failure.

Data obtained through bond strength testing was tabulated using statistical program for social science (SPSS version 21, Inc., Chicago, US). Normality of data obtained was assessed using Kolmogorov-Smirnov test. Means and standard deviations were compared using analysis of variance (ANOVA) and Tukey’s post hoc test at a significance level of (p < 0.05).

## RESULTS

The highest mean SBS was observed in Group-4 ECL-C (21.55±3.08). The lowest mean SBS was displayed in Group-3 ECL-ME (14.25±3.55). Mean SBS values of Group-4 ECL-C (21.55±3.08) and Group-2 ECL-V (20.74±4.15) were found to be comparable (p <0.05). Similarly, SBS values of Group-1 ECL-BA (15.48±3.62) and Group-3 ECL-ME (14.25±3.55) were comparable (p <0.05). For bond strength values, analysis of variance (ANOVA) showed significant difference among the study groups (p<0.001) (Table-II).

**Table-II:** Means and SD for bond strength among different study groups using ANOVA and Tukey multiple comparisons test.

Surface treatment /type of cement used in experimental groups	Mean ± SD (Mpa)	P-value!
Group-1 ECL-BA *	15.48±3.62	<0.001
Group-2 ECL-V ≠	20.74±4.15	
Group-3 ECL-ME *	14.25±3.55	
Group-4 ECL-C ≠	21.55±3.08	

ECL: Er,Cr:YSGG laser, BA: Bioactive Cement, V: Variolink II, ME: Maxcem Elite, C: Calibra The highest and lowest SBS values are in bold, ≠ Significantly different from groups- ECL-BA, ECL-ME (p <0.05),

* Significantly different from groups- ECL-V, ECL-C (p <0.05), (Tukey multiple comparison test), ! Showing significant difference among study group (ANOVA).

In relation to failure modes of specimens, adhesive failure dominated among experimental groups. In Group-4 ECL-C and Group-2 ECL-V admixed mode of failure was more prevalent. Whereas, adhesive failure mode was persistent in Group-3 ECL-ME and Group-1 ECL-BA (Table-III).

**Table-III:** Modes of failure among different experimental groups

Experimental groups	Adhesive (%)	Cohesive (%)	Admixed (%)
Group-1 ECL-BA	90	10	-
Group-2 ECL-V	20	10	70
Group-3 ECL-ME	70	20	10
Group-4 ECL-C	30	10	60

ECL: Er,Cr:YSGG laser, BA: Bioactive Cement, V: Variolink II, ME: Maxcem Elite, C: Calibra.

## DISCUSSION

The present study was based on the hypothesis that dentine surface etched with ECL and bonded to BA cement will exhibit better SBS score to conventional dual cure cements. Remarkably, the hypothesis was rejected as dentine lased with ECL and bonded to conventional cement C and V displayed better SBS values. However, it was noteworthy that SBS values of ME and BA cement were comparable bonded to photosensitized dentine using ECL.

ECL is now widely used in dentistry for various dental applications.^7^ ECL conditioning of dentine works by ablating the dentinal surface, as it is better absorbed by water, collagen and hydroxyapatite.^13^ ECL on the dentine opens the odontoblastic tubules, reveals surface free of smear layer leading to flaking and peritubular cuffing.^14^ Consequently, the process is also explained by Lin et al., that laser ablation on dentine results in micro explosion within the inorganic part of dentine hence improving bond integrity.^15^ However, processes related to the laser preparation was not part of the current study.

In the present study all the dentinal surface was leased by ECL at (30Hz and 4.5W) as the process is less technique sensitive, requires less chair time and can be used in a moist environment.^14^ Bond strength values were evaluated using SBS test in a universal testing machine to follow standardization, homogeneity and consistency.^16^ SBS of two cements V (20.74±4.15) and C (21.55±3.08) were found to be comparable and better than BA cement. A possible explanation for high SBS scores in these two groups can be attributed to the presence of silane coupling agent (BISGMA) in C and (UDMA) and (TEGDMA) in V cements which might have improved dentine adhesion.^17^ Secondly, the adhesive system used in the present study was Tetric N Bond i.e., (fifth generation bonding agent) which is ethanol based containing small amount of water.^18^ The extrinsic water in adhesive agent along with intrinsic moisture during photo ablation of dentine surface with laser, caused rehydration of the dentinal collapsed collagen matrix hence improving bond integrity.^18^ Moreover, in the authors opinion, Tetric N bond adhesive is a combination matrix of both hydrophilic and hydrophobic monomers and intermediate (bis-GMA) nature.^19^ This characteristic of adhesive allows bridging of gap between the hydrophilic tooth and hydrophobic resin cement in diverse conditions which indirectly improves SBS scores. Furthermore, bond strength between resin cement and dentine is dependent on multiple factors. These may range from hybrid layer thickness, number and length of resin tags, surface roughness, void formation and integrity of interface.^20^

The lowest SBS values were displayed by ME cement (14.25±3.55). A possible explanation for this observation, is that weak acid capability of one step self-etch cement and poor penetration of monomers in resin tags. Furthermore, shallower and gentler pattern on dentine surface by ME can be credited to low bond strength scores in this group.^21^ In the present study, a polyacrylic acid conditioner (Ketac conditioner) was applied to ECL treated dentine. Polyacrylic acid conditioner is recommended to remove smear layer and facilitate formation of a hybrid layer to augment the adhesive bond integrity.^22^ However, application of polyacrylic acid conditioner failed to show significant improvement of SBS in the presence of phototherapy. A possible explanation for this observation, is the impact of phototherapy (Er,Cr:YSGG) on dentinal tubule viability and removal of smear and resulting in a susceptible dentine surface for adhesive bonding (free of smear layer).^23^ Therefore, the authors do not recommend the clinical use of Ketac conditioner (polyacrylic acid) in the surface conditioning of phototherapy treated dentine when bonded to different cement types.

Interestingly, BA cement showed SBS values (15.48±3.62) which were comparable to ME cement (14.25±3.55). A plausible explanation is the absence of silane coupling agent in BA cement. The manufacturers claim no Bisphenol A, No BisGMA, no BPA derivatives in the cement itself.^24^ Secondly, in the authors belief poor affinity of bioactive glass particles in BA cement to dentinal collagen may attribute to poor bond strength values. Moreover, since it’s a bioactive cement and it gains strength by exchange of ions from saliva and oral environment, present in-vitro study design may have limited its efficacy and effectiveness compromising bond strength values of BA cement. The authors can only speculate the possibilities of low bond strength values in this group as there are no studies done to extrapolate similar findings.

In relation to modes of failure admixed type of failure were dominant in ECL-V and in group ECL-C. Thermomechanical ablation of Er,Cr:YSGG on dentinal surface compromising the physical properties may be a reason for this type of failure. Furthermore, lateral forces, type of cement and debonding method may influence admixed type of failure. However, ECL-BA and ECL-ME presented adhesive type failure. These findings correspond to SBS values of these cement. A possible clinical implication of these results indicates that Er,Cr:YSGG laser has a potential to condition dentin surface bonded to BA and other conventional esthetic resin cements (Calibra, Variolink and Maxcem Elite).

### Limitation of the study

These are based on its in-vitro study design. These results are only applicable on the type of cement used, frequency and power of Er,Cr:YSGG laser, type of bonding agent, curing technique and dentine structure. In addition, the results of in-vitro studies cannot be applied on clinical setting henceforth long-term clinical studies are recommended. Future studies, should be directed on surface profilometry of dentine surface lased with Er,Cr:YSGG. Nevertheless, the use of any resin cement should be dependent on the clinical situation and operator’s judgment.

## CONCLUSION

Bioactive cement when bonded to dentine surface conditioned with Er,Cr:YSGG laser showed lower SBS in comparison to conventional esthetic resin cements (Calibra and Variolink). Therefore, studies assessing other effective surface treatments for the adhesive bonding of Bioactive cements to tooth dentine are recommended.

### Author`s Contribution:

**FAK:** Data collection, study design, manuscript writing, final manuscript approval, is responsible for integrity of research.

**SA:** Final corrections done, Manuscript design and editing, Manuscript approval

**MN:** Data collection, study design, manuscript drafting, data analysis, manuscript approval.

**MBS and MN:** Data collection, manuscript approval and data interpretation.

**FV:** Data collection, writing, revise, editing and final manuscript approval.
